# On-Surface Synthesis and Characterization of Radical Spins in Kagome Graphene

**DOI:** 10.1021/acsnano.4c15519

**Published:** 2025-01-10

**Authors:** Rémy Pawlak, Khalid N. Anindya, Outhmane Chahib, Jung-Ching Liu, Paul Hiret, Laurent Marot, Vincent Luzet, Frank Palmino, Frédéric Chérioux, Alain Rochefort, Ernst Meyer

**Affiliations:** † Department of Physics, University of Basel, Klingelbergstrasse 82, Basel 4056, Switzerland; ‡ Engineering Physics Department, Polytechnique Montréal, Montréal (Québec) H3C 3A7, Canada; § Université de Franche-Comté, FEMTO-ST, CNRS, Besançon F-25000, France

**Keywords:** Kagome graphene, on-surface synthesis, radical spins, scanning tunneling microscopy, atomic force microscopy, density functional theory

## Abstract

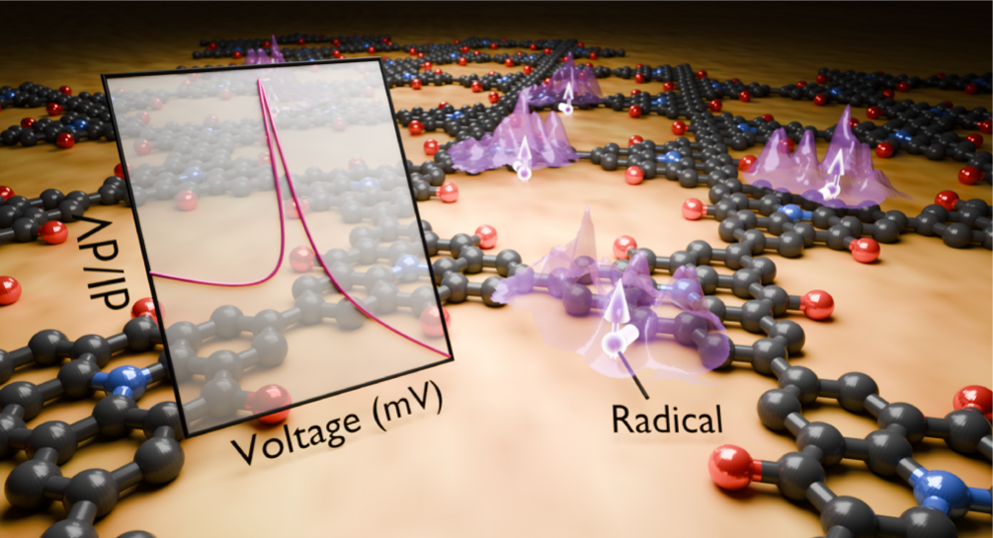

Flat bands in Kagome graphene might host strong electron correlations and frustrated magnetism upon electronic doping. However, the porous nature of Kagome graphene opens a semiconducting gap due to quantum confinement, preventing its fine-tuning by electrostatic gates. Here we induce zero-energy states into a semiconducting Kagome graphene by inserting π-radicals at selected locations. We utilize the on-surface reaction of tribromotrioxoazatriangulene molecules to synthesize carbonyl-functionalized Kagome graphene on Au(111), thereafter modified *in situ* by exposure to atomic hydrogen. Atomic force microscopy and tunneling spectroscopy unveil the stepwise chemical transformation of the carbonyl groups into radicals, which creates local magnetic defects of spin state *S* = 1/2 and zero-energy states as confirmed by density functional theory. The ability to imprint local magnetic moments opens up prospects to study the interplay between topology, magnetism, and electron correlation in Kagome graphene.

## Introduction

Kagome graphene (KG),^1−3^ a two-dimensional (2D) arrangement of corner-sharing graphene triangles, is long regarded as an ideal candidate for strongly correlated electron phenomena and frustrated magnetism.^4−9^ Its electronic band structure features van Hove singularities (vHSs), Dirac cones, and nontrivial flat bands,^5^ whose filling could be controlled by tuning of the chemical potential using electrostatic gates.^10,11^ Geometrical frustration of Kagome lattices might also cause magnetic frustration as exemplary shown in Kagome metals,^12−14^ making Kagome materials prime candidates for the realization of quantum-spin-liquid states and fractionalized excitations.^15,16^ However, the synthesis of atomically precise Kagome graphene into pure and extended sheets remains a challenging task, while its integration into a gate-tunable device has yet to be realized.

On-surface synthesis under ultrahigh vacuum (UHV)^17^ has become an exciting alternative to lithography or plasma etching methods for fabricating low-dimensional nanographenes with atomic precision,^18−20^ structure and electronic properties of which are investigated at the atomic scale with scanning tunneling microscopy (STM) and atomic force microscopy (AFM).^21−23^ The rise of carbon-based magnetism in open-shell nanographene^24^ has further expanded the potential applications of nanographenes to spintronics and quantum technologies, as demonstrated by triangulene derivatives^25−29^ and few other precursors^30−32^ through the observation of singly occupied/unoccupied molecular orbitals (SOMOs/SUMOs),^33^ Kondo resonances,^26^ and spin-flip excitations using scanning tunneling spectroscopy (STS).^34,35^ In two dimensions, covalent networks with the Kagome structure were also produced on noble metals using on-surface chemistry,^1−3^ making them potentially suitable for a transfer into gate-tunable devices.^36^ However, their porous character inherently induces large semiconducting gaps due to quantum confinement and heteroatom doping,^37^ which shift their flat bands to high energies away from the Fermi level *E*_F_, thus preventing a fine-tuning with electrostatic gates. To address this issue, we aim to reproduce the conceptual approach introduced by Rizzo et al. for semiconducting GNRs^38−40^ consisting in introducing zero-mode states through the periodic incorporation of radical sites in the KG structure. These low-energy states offer the opportunity to investigate the interplay between topology, magnetism, and electron correlation in Kagome graphene when directly adsorbed on a metal or in nanoscale devices.

**Figure 1:**
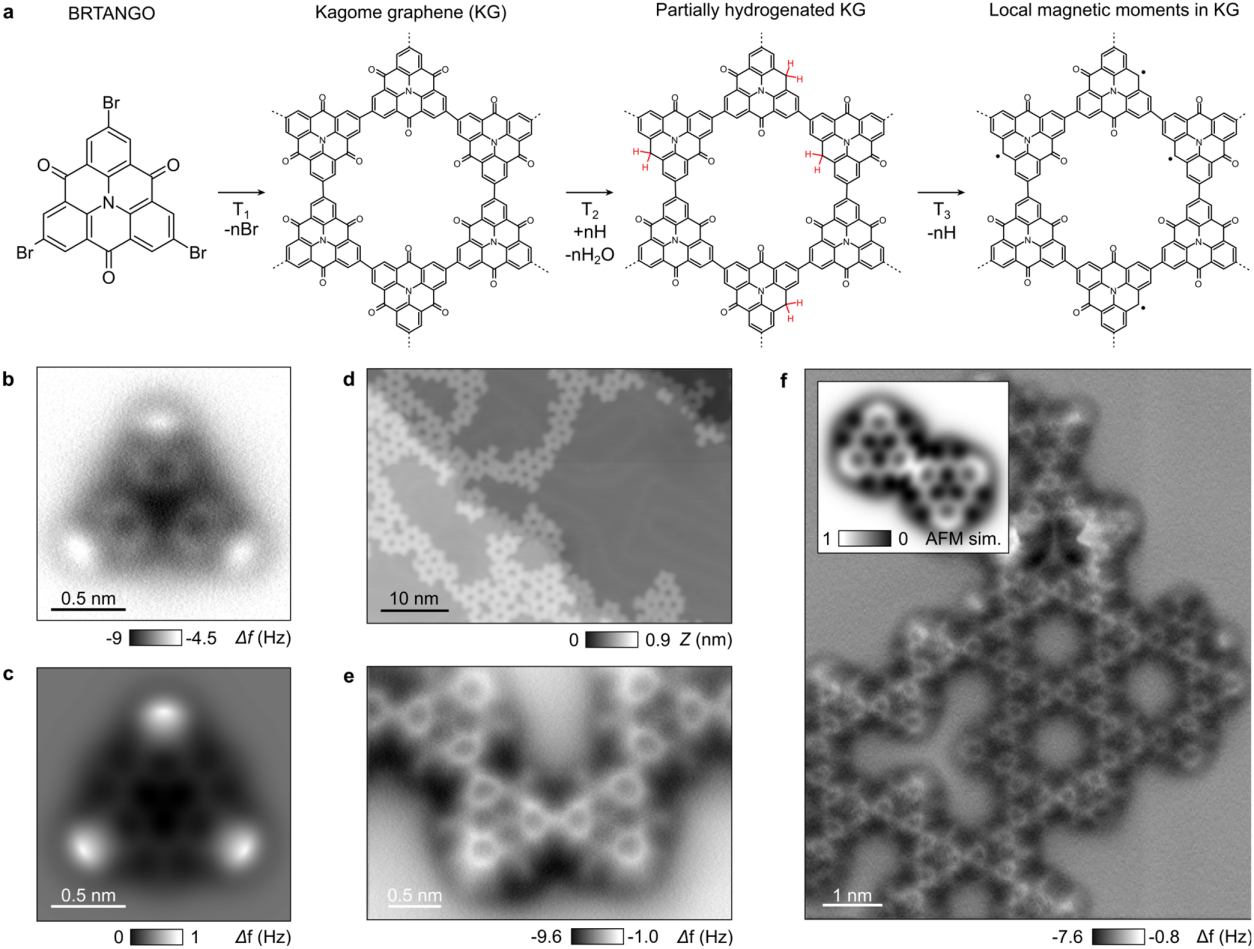
Hierarchical synthesis of magnetic radicals in carbonyl-functionalized Kagome graphene by an on-surface reaction. (a) Chemical structure of the tribromotrioxoazatriangulene (BRTANGO) molecule. (b) AFM image with a CO-terminated tip of the isolated BRTANGO precursor (*I*_t_ = 1 pA, *V*_s_ = 0.25 V). (c) Corresponding AFM image simulation. (d) STM image of the Kagome graphene after annealing the substrate at 450 K (*I*_t_ = 1 pA, *V*_s_ = 0.05 V). (e)–(f) AFM image of the chemical structure of carbonyl-functionalized Kagome graphene, revealing the covalent coupling between azatriangulene monomers. The inset of **f** shows a simulated AFM image for a covalent dimer.

Here, we examine using STM and AFM at low temperature a synthetic strategy to create radical sites from carbonyl- (C = O)-functionalized Kagome graphene (KG) (Figure 1a). We use the on-surface reactions of tribromotrioxoazatriangulene (BRTANGO) molecules on a Au(111) surface under UHV conditions,^1,2^ in which we reduce the peripheral carbonyl groups to CH_2_ end groups through their exposure to atomic hydrogen produced by an ion gun^28,33,41^ or a plasma source. A subsequent thermally activated dehydrogenation step transforms these groups into C–H radicals embedded into the Kagome graphene lattice, which is confirmed by a Kondo resonance in tunneling spectra and DFT calculations. Although the complete reduction of the KG polymer is experimentally limited by the on-surface reaction, our results demonstrate a reasonable strategy to engineer localized zero-energy states in otherwise semiconducting porous nanographene and might serve as a first step for the synthesis of a fully metallic Kagome graphene.

## Results and Discussion

### Electronic Structure of the Carbonyl-Functionalized Kagome Graphene

BRTANGO molecules were sublimated from a Knudsen cell in UHV on the substrate kept at room temperature, leading to the formation of self-assembled molecular domains (see Methods, Supporting Figure 1). The AFM image of the isolated precursor and its simulation using the probe-particle model^42^ (Figure 1b,c) shows the peripheral bromide atoms attached to the corners of the azatriangulene as bright protrusions, while carbonyl side groups appear as dark contrast at edges of the molecule. Annealing the gold substrate to *T*_1_ = 450 K initiates the Ullmann coupling reaction, leading to extended domains of Kagome graphene as shown in Figure 1.^1,2^Figure 1d–f shows representative AFM images of the chemical structure, unveiling the newly formed C-C bonds between azatriangulene monomers. The AFM contrast also distinguishes carbonyl side groups as faint lines attached to the sides of triangulene. They also appear darker than the intermolecular C-C bonds, in relative agreement with the simulated AFM image shown in the inset of Figure 1f. Note finally that, in both the isolated BRTANGO precursor and its polymerized counterpart (Figure 1b,e), the central nitrogen of the molecule always shows a darker contrast than the neighboring carbon atoms.

**Figure 2:**
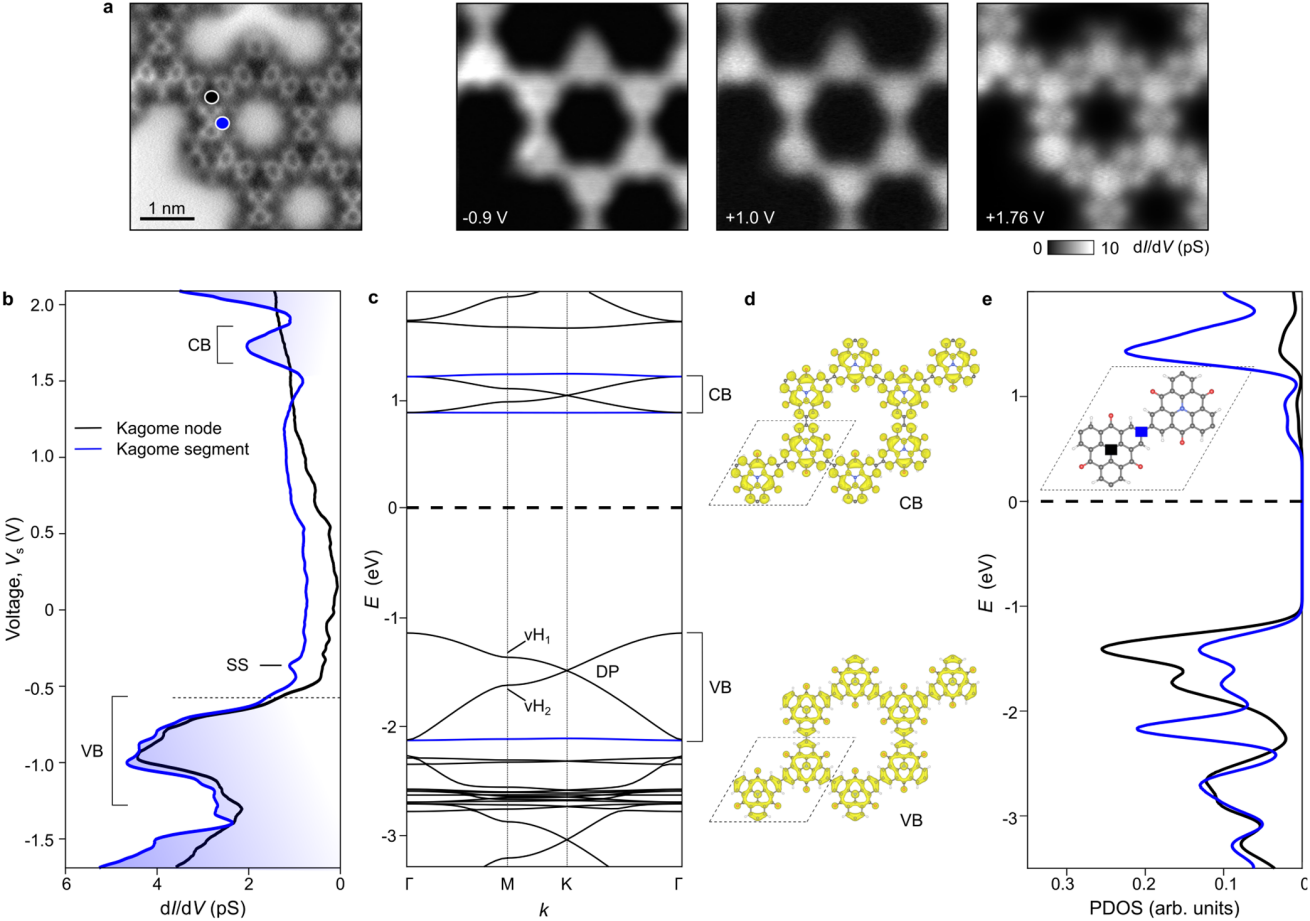
Electronic structure of carbonyl-functionalized Kagome graphene. (a) AFM image of a Kagome pore and a series of spatial d*I*/d*V* maps recorded at *V*_s_ = −0.9 +1.0, and +1.76 V, respectively. (b) Site-dependent d*I*/d*V* spectra acquired at the Kagome node (black) and at the Kagome segment (blue) (*A*_mod_ = 20 mV, *f*_0_ = 611 Hz). VB and CB correspond to the onset of the valence band (−0.6 eV) and conduction band (+1 eV), while SS refers to the confined surface state of Au(111) in the KG pore. (c) KG band structure calculated by DFT + *U* revealing flat conduction bands near 1 eV (blue lines). DP refers to the Dirac point, and vH_1_ and vH_2_ are van Hove singularities. (d) Frontier orbitals of the KG structure at the CB and VB edges. (e) Projected density of states (PDOS) extracted at the azatriangulene center (black) and at one carbon atom of the segment (blue) (see inset).

To gain insight into the KG electronic structure, we acquired site-dependent differential conductance (d*I*/d*V*) measurements at 4.4 K (Figure 2). By comparing the spectra acquired at the central nitrogen atom (black dots in Figure 2a) with the segment of the KG lattice (blue), we assign the valence band edge (VBE) and conduction band edge (CBE) to −0.6 and 1.6 eV, corresponding to a band gap of about 2.2 eV. This observation confirms the semiconducting character of KG polymer as previously reported.^2,6^ The resonance at ≈ −450 mV is only observed near KG segments (blue spectra) and is attributed to the confinement of the Au(111) Shockley surface state (SS) by the KG pore. The resonance at +1.75 V is also localized to segments since it disappears when the azatriangulene center is probed (black spectra). This is corroborated by the series of spatial d*I*/d*V* maps presented in Figure 2a, revealing the appearance of two lobes at segments for *V*_s_ = 1.76 V, which disappears for the map at VB (*V*_s_ = −0.9 V) or within the gap (*V*_s_ = +1.0 V).

Using density functional theory (DFT) calculations with Hubbard *U* corrections (see Methods), we calculated the band structure of the freestanding KG (Figure 2c) showing the characteristic features of a Kagome graphene: (1) the presence of Dirac cones (DC) and van Hove singularities (vH) due to π-orbitals delocalized across the hexagonal carbon lattice; (2) a series of flat bands inherited from the Kagome geometry. The Dirac points emerge at the *K*-point at about −1.5–3.1, and +1.0 eV, respectively. Three flat bands plotted as blue lines in Figure 2c are positioned at −2.1, 0.9, and 1.2 eV, respectively. For a better comparison with the d*I*/d*V* measurements, Figure 2e also shows the projected density of states (PDOS) extracted at nodes (black) and segments (blue), respectively.

At +1 eV, the Dirac cone sandwiched by two flat bands (Figure 2c) emerges as a broad resonance in the corresponding PDOS (blue spectra of Figure 2e), which disappears at nodes of the KG structure (black spectra). Both the theoretical line shape and its localization are in agreement with the d*I*/d*V* spectra of Figure 2b, allowing us to ascribe the resonance at +1.75 V to their experimental signature. Similar to the experimental d*I*/d*V* map of Figure 2a, the frontier orbitals obtained by DFT + *U* (CB of Figure 2d) also show an increase of localization of the density of states (LDOS) at KG segments and a reduction at the central nitrogen of the azatriangulene.

At −1 eV below *E*_F_, the PDOS curves show a series of resonances with higher weight at nodes than segments which we relate to the region including the Dirac cone, a flat band, and the two van Hove singularities (Figure 2c). Although the line shape of the experimental spectra at *V*_s_ ≤ – 0.5 V appears similar to that, this subtle variation in intensity is not experimentally captured, likely due to the hybridization of the KG DOS with Au states.

### Hydrogenation of Carbonyl Groups and Radical’s Formation

To hydrogenate peripheral C=O groups, we exposed the KG/Au(111) held at room temperature in UHV to atomic hydrogen produced using either a hydrogen cracker or a plasma source (see Methods).^28,33^ The reduction reaction transforms carbonyl side groups of azatriangulene monomers into sp^3^-hybridized carbon atoms (CH_2_ groups colored in red in Figure 1a). Reacted azatriangulene monomers turn slightly three-dimensional due to the formation of the bulky CH_2_ groups at their sides.^33^ Supporting Supporting Figure 3a–b show a series high-resolution STM/AFM images of the hydrogenated KG lattice after such a reaction. While in the STM topographic image the formed CH_2_ groups can be difficult to identify, they become apparent in AFM images since the AFM contrast is sensitive to three-dimensional relaxations of molecules (see yellow arrows in Supporting Figure 3b). Supporting Figure 3c further shows close-up AFM images of a monomer with a single CH_2_ side group, along with a bond-resolved STM (BRSTM) image.

**Figure 3:**
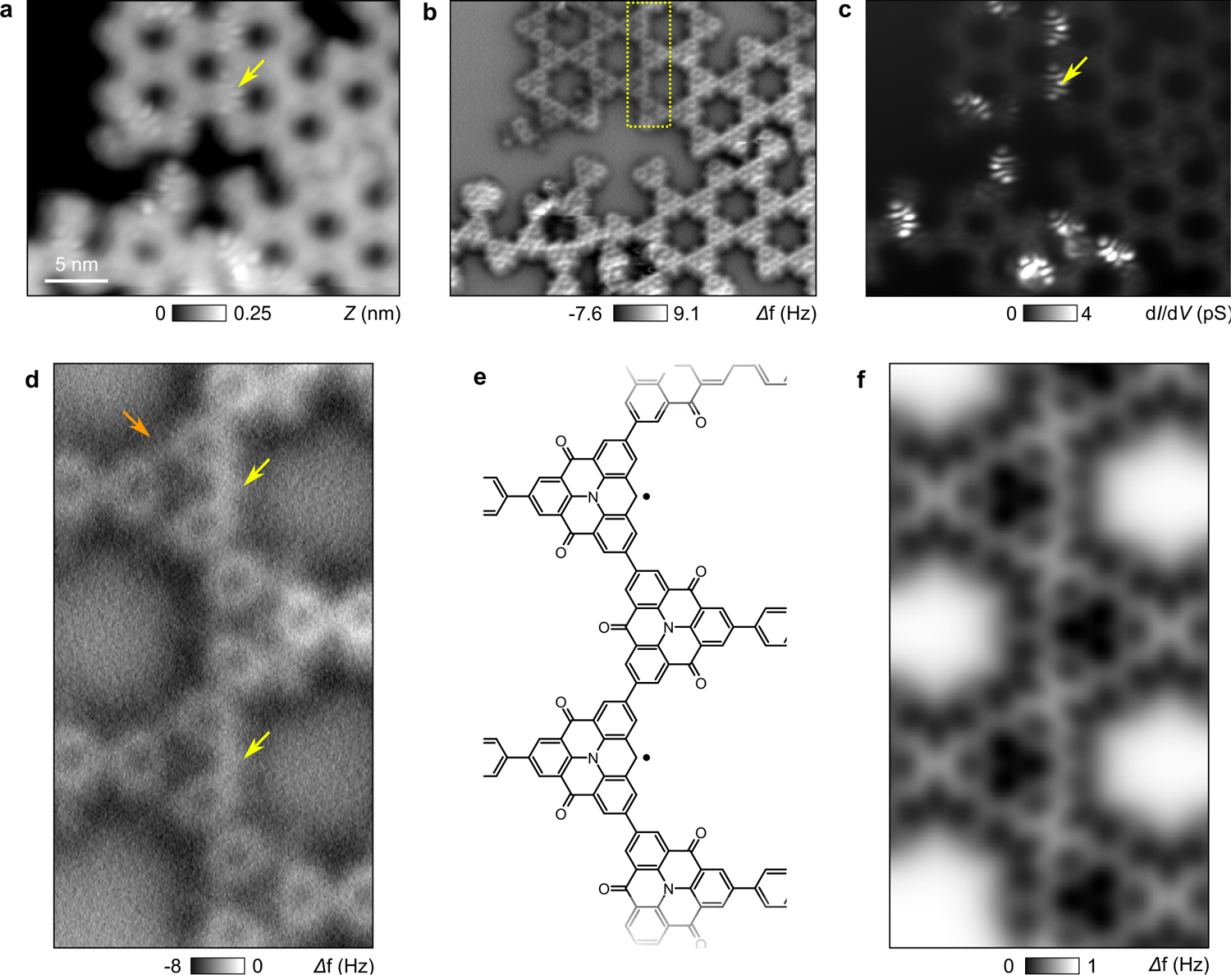
Structural characterization of radical sites by STM/AFM. (a) STM overview image of the KG after dehydrogenation of CH_2_ groups (*I*_t_ = 1 pA, *V*_s_ = 0.05 V). (b) Corresponding AFM image with a CO-terminated tip (*f*_0_ = 26 kHz, *A*_osc_ = 50 pm). (c) Spatial d*I*/d*V* maps acquired at zero-energy. Bright features (yellow arrow) correspond to the zero-energy modes arising from the presence of π-radicals of the KG. (d) Close-up AFM image distinguishing carbonyl side groups (orange arrow) and π-radicals (yellow arrows). (e) Deduced chemical structure in gas-phase and (f) the simulated AFM image.

The synthesis of π-radicals is finally obtained by annealing the substrate to *T*_3_ = 470–500 K leading to the partial dehydrogenation of the CH_2_ side groups into CH radicals (Figure 1a). Figure 3a,b display representative STM and AFM images of the KG structure after the radical’s formation. At low bias value (*V*_s_ ≈ 15 mV), STM images reveal bright features superimposed to a few monomers as exemplarily shown by a yellow arrow in Figure 3a. As compared to the partially hydrogenated KG (Supporting Figure 3), AFM images now show only planar monomers, even for those with bright STM features. The absence of any geometrical relaxations of the molecules thus indicates the successful dehydrogenation of the CH_2_ side groups and the presence of radical CH sites. This observation is further corroborated by zero-energy d*I*/d*V* maps (Figure 3c), which reflects the spatial distribution of the zero-energy states. Similar for all the reacted monomers, this spatial signature is analogous to that of Kondo resonances for π-electrons in nanographene.^28,33^

We next characterized in more detail the AFM contrast of these radicals. Figure 3d–f presents an AFM close-up where two of the monomers exhibit zero-energy states in the low-voltage STM image of Figure 3 (dashed rectangle). Radicals appear by AFM as bright lines at one side of the monomer (yellow arrows of Figure 3d), which contrast with the darker contrast of the carbonyl groups (orange arrow). By assuming the chemical structure based on the AFM image (Figure 3e), Figure 3f shows the simulated AFM image using the probe-particle model. The good agreement between the model and experimental data allows us to correlate the observation of zero-energy states to the presence of π-radicals in the KG structure.

The reduction of carbonyls into CH radical sites is obtained by a two-step reaction, which requires (1) the hydrogenation of carbonyl groups by exposure to atomic hydrogen in ultrahigh vacuum and (2) their reduction from CH_2_ groups into CH radical sites by annealing of the substrate (Figure 1). To estimate it, we systematically characterized large areas of the KG/Au(111) sample by STM/AFM. While each monomer contains three carbonyl groups, we mainly observed using the hydrogen cracker the reduction of one carbonyl per molecules (Figure 3c) and never reached a reaction yield superior (defined as η the number of reacted molecules over the total number of molecules) to η = 11–15%. Using the low-temperature hydrogen plasma (see Methods, Supporting Figures 4 and 5), the density of radicals can be increased by longer plasma exposure up to η = 40–50% without any disruption of the lattice (Supporting Information, Figure 5c). We also emphasize that long exposure time using a cracker source (see Methods) is not possible when the sample directly faces the hot filament of the source as it introduces surface defects, preventing further experimental characterization. However, the cracker source has been shown to lead to the full hydrogenation of nanographenes as reported here,^32^ probably by exposing only the backface during several hours. In comparison, the plasma treatment has the advantage to have a higher density of atomic hydrogen requiring only a few minutes of exposure to achieve a full hydrogenation reaction. However, as shown in Supporting Figure 5d–f, the complete reduction of the KG polymer initiated by annealing the substrate is systematically accompanied by the disruption of the kagome lattice. We think that the stress accumulated by the structure during the dehydrogenation reaction prevents its full reduction, as will be discussed below.

### Kondo Resonance From Radical Sites in Tunneling Spectroscopy

**Figure 4:**
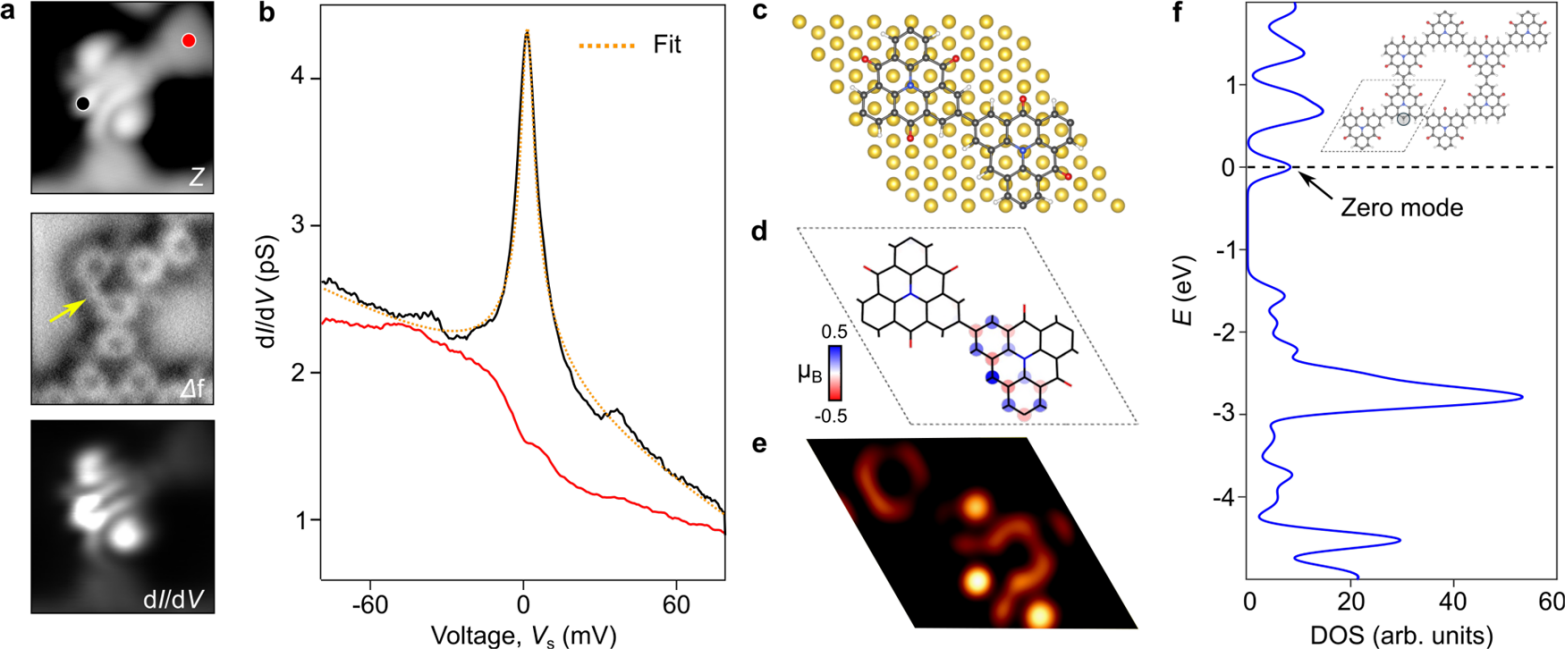
Kondo resonance from the radical sites. (a) STM topography, AFM image and zero-energy d*I*/d*V* map of a reacted monomer. (b) Low-energy d*I*/d*V* spectra acquired at the black and red dots in **a** corresponding to a reacted (black) and an unreacted monomer (red), respectively. Lock-in amplitude: 1.5 mV; *f*_0_ = 611 Hz. A Hurwitz-Fano line shape^43^ is used to fit the zero-bias peak, which we attribute to a Kondo resonance from the spin-1/2 π-radical. (c) Structure of a dimer on Au with one radical site per unit cell (UC) and (d) spin density. (e) Simulation of the STM image using the Tersoff–Hamman approximation. (f) Freestanding density of states (DOS) calculated by DFT + *U* showing the zero-energy states induced by a single radical in the KG (black dots in the inset).

To characterize the magnetic signature of radical sites, we performed low-energy d*I*/d*V* point-spectra at *T* = 4.5 K. Figure 4a shows the STM topographic image and AFM image of a reacted monomer, together with its zero-energy d*I*/d*V* map. The yellow arrow points to the position of the radical site. In Figure 4b, we plot representative d*I*/d*V* point-spectra recorded at the radical (black) as compared to a pristine molecule (red), which positions are shown in Figure 4a. The zero-bias peak observed at the radical site, absent for the unreacted monomer, is attributed to a Kondo resonance from its spin 1/2 state.^26^ Its line width (fwhm ≤7.2 mV), extracted from fitting several experimental spectra with the Hurwitz-Fano line shape,^43^ is consistent with a Kondo temperature of approximately 21–25 K. Note also that, for such low radical density, the radical spin are too far apart to interact (Figure 3c). Thus, no signature of spin excitations has been observed in d*I*/d*V* spectra similar to those of references.^31−35^

We further explored the electronic structure of the KG using DFT + *U*. The relaxed structure of the unit cell (UC) is shown in Figure 4c, which consists of a dimer with one radical adsorbed on Au(111) (black circle in the inset of Figure 4f). The calculated nonspin-polarized density of states (DOS) for the freestanding case shows a nonbonding zero-energy state at *E*_F_, indicating a high-spin state of the KG lattice. Additionally, we observe the opening of a Coulomb gap in the spin-polarized DOS of Supporting Figure 6, representing the ground state of the system. This gap results from the repulsive energy cost associated with unpaired spins attempting to occupy the same site in this magnetic system.^44^ The theoretical spin-density map of the dimer (Figure 4d) shows magnetic moments localized along the radical site but absent from the neighboring carbonyl groups. The calculated LDOS map shows the localized nature of the unpaired electron (Figure 4e), which is in good agreement with the d*I*/d*V* map of Figure 4a. Using a 2 × 2 supercell, we also estimated the Heisenberg spin exchange parameter^45^ (*J* = *E*_AF_ – *E*^_FM_^ (triplet) ≈ 0 meV) indicating its paramagnetic nature.

### Beyond Experiments: The Influence of Radicals on Local Magnetism

**Figure 5:**
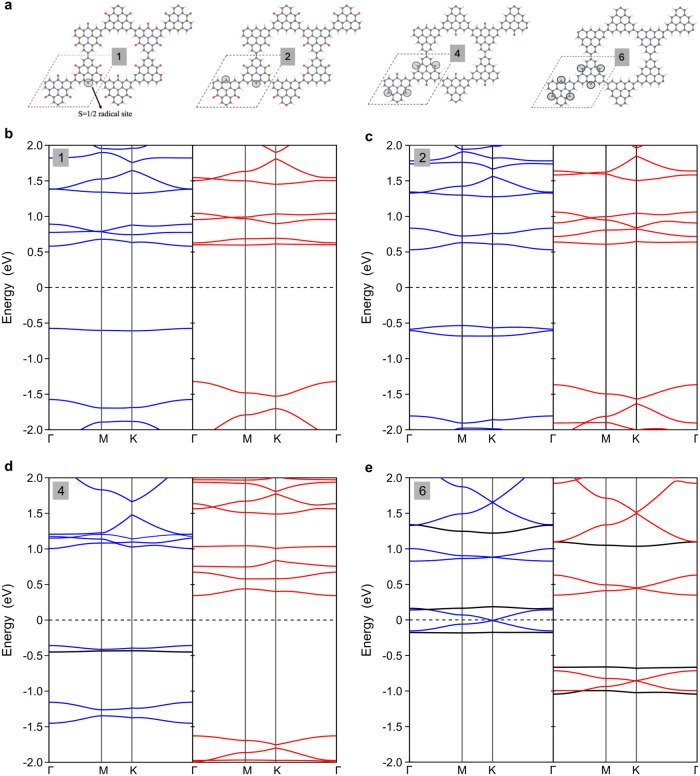
Influence of the radical density on the KG band structure. (a) Structural configurations of the KG with one, two, four, and six radicals per unit cell, respectively. (b)–(e), Calculated band structures in their ferromagnetic (FM) states, showing α (blue) and β (red) spin channels. The figure highlights the impact of radical concentration on spin polarization, symmetry breaking, and the disappearance and reformation of Dirac cones and flat bands (black lines in (d) and (e)).

Although a complete reduction of carbonyls in the KG structure has not been achieved experimentally, we extended our theoretical investigation using DFT to the influence of multiple radicals on the magnetic properties of the KG unit cell (UC). Starting with two and four radicals per UC (denoted 2 and 4 in Figure 5a), a clear antiferromagnetic (AF) coupling between the spin sites of neighboring monomers is observed (Supporting Figure 7b–c). The energy differences between the AF and the ferromagnetic (FM) ground state are indicated in Supporting Figure 7, highlighting the system’s energetic preferences. In the case of four radicals, the system displays FM coupling between spin sites of each monomer while maintaining a collective AF coupling between neighboring monomers. This magnetic behavior mirrors that of the triangulene dimer system, which also features four unpaired electrons and exhibits similar magnetic properties.^44^

### DFT Calculations of Jahn–Teller Distortion for Fully Reacted Monomers

Now considering six radicals per UC (Figure 5a and Supporting Figure 7d), an unexpected deviation of the spin configuration is observed. Instead of a *S* = 3/2 spin state per monomer resulting from the three radicals, a Jahn–Teller distortion reduces the spin state to *S* = 1/2 per monomer.^28^ This distortion lifts the degeneracy of the electronic configuration, stabilizing the system by lowering its overall energy. Within individual monomers, AF coupling occurs between adjacent atoms, ensuring that the system adheres to Ovchinnikov’s rule,^46^ which requires alternating spin states between adjacent atoms in organic conjugated systems. However, the small energy difference of 4.5 meV between the AF and FM states suggests a very subtle preference for the FM configuration between monomers, although the system would remain paramagnetic at certain temperatures or under external conditions. This subtle balance between FM and AF interactions with multiple radicals underscores the significant role played by structural distortions (i.e., the Jahn–Teller effect) in modulating the system’s magnetic properties. This structural relaxation is manifesting as a shortening of the C-N bonds at the center of the azatriangulene monomer in a *S* = 1/2 spin state compared with the *S* = 3/2 counterpart (Supporting Figure 8). Despite having six free radicals per UC and the potential for higher spin states, the reduction to a lower spin state accompanied by an AF coupling between neighboring atoms per monomers demonstrate the robustness of Ovchinnikov’s rule. Experimentally, the fully reacted monomer embedded into the KG lattice was never probed by STM as the dehydrogenation reaction activated by annealing the substrate systematically destroyed the structure (Supporting Figure 5). We think that the Jahn–Teller effect and the structural distortions associated with it may be responsible for the high stress accumulated on the KG polymer, leading to its rupture during the reaction. As in ref (32), the dehydrogenation of the hydrogenated KG structure assisted by local voltage pulses from the STM tip could solve this problem. This will be addressed in future experiments.

### Revival of Dirac Cones and Kagome Flat Bands Predicted by DFT

Figure 5b–e presents the band structures for the configurations with one, two, four, and six radicals per UC in their ferromagnetic (FM) state, respectively. Although the two- and four-cases have antiferromagnetic (AF) ground states, we focus on their FM states for a more consistent comparison. As the number of radicals per UC increases, the figure shows how the radical concentration affects spin polarization, symmetry breaking, and electronic band structure, with particular attention to the survival of Dirac cones and flat bands inherited from the Kagome lattice. For one radical per UC (Figure 5b), the band structure shows distinct spin-up (blue) and spin-down (red) channels, indicating clear spin polarization caused by the paramagnetic ordering. The introduction of a single radical site disturbs the symmetry of the pristine Kagome lattice, causing the characteristic Dirac cones at the *K*-point of the Brillouin zone (BZ) and the Kagome flat bands to disappear. Despite this break in symmetry, electronic bands are still dispersive around *E*_F_, indicating a certain level of electronic mobility. The moderate splitting of the spin channels suggests that this configuration only weakly localizes the electronic states maintaining a relatively conductive behavior.

For two radicals per UC (Figure 5c), we observe the resurgence of Dirac cones, as the interaction between the two radicals reintroduces some symmetry compensation in the configuration we have chosen (2 in Figure 5a). The bands near *E*_F_ are slightly dispersive, indicating reduced localization. In contrast, four radicals per UC show a pronounced localization (Figure 5d) as evidenced by the appearance of flat bands at about −0.5 eV below *E*_F_, while Dirac cones have disappeared due to the reduction in the symmetry of the system. The flattening of the bands near *E*_F_ implies that the electronic states are strongly localized at the radical sites and that the electronic properties of the system are dominated by the interactions between them and by symmetry breaking. Introducing six radicals per UC, which corresponds to the fully reduced KG (6 in Figure 5a), restores the symmetry of the Kagome lattice. This is reflected in the band structure of Figure 5e by the resurgence of Dirac cones at the *K*-point, which are more dispersive than previous cases, a signature of a Dirac-like system with high electronic mobility. This observation is also a remarkable departure from the trend of localization seen in the two- and four-radical systems. Although the Jahn–Teller distortion reduces the expected spin state of each monomer from *S* = 3/2 to 1/2 in this case, it does not prevent the overall system from regaining its symmetry. This behavior indicates that the fully reacted system not only recovers the KG fingerprints but also exhibits high-mobility electronic states, in stark contrast with the highly localized states of the four-radical system (Figure 5d).

Importantly, the Dirac cone near *E*_F_ for six radicals per UC is sandwiched by KG flat bands (black lines in Figure 5e), which are now shifted closer to *E*_F_ compared to the pristine KG structure. Although this configuration leads to a spin-polarized ground state, we compare this in Supporting Figure 9 its diamagnetic properties (blue in Supporting Figure 9a) with its pristine counterpart (Figure 3). The reduction of all carbonyl side groups to radicals not only restores the key Kagome features but also leads to their emergence near *E*_F_ due to the changes in local potential induced by the reduction reaction. Additionally, Jahn–Teller distortion contributes to stabilizing these localized states, pushing them closer to *E*_F_. Therefore, the resulting DOS for the fully reduced configuration (blue in Supporting Figure 9b) is now metallic as it possesses zero-mode states. This is in stark contrast with the semiconducting character of the pristine Kagome lattice (red in Supporting Figure 9b), in which flat bands lie at about 1 eV above the Fermi level.

## Conclusions

In summary, we investigated by means of high-resolution AFM a synthetic route to produce radical sites in a carbonyl-functionalized KG and demonstrated their magnetic signature by tunneling spectroscopy and DFT. The synthesis of KG was obtained by the on-surface reaction of tribromotrioxoazatriangulene (BRTANGO) molecules on a Au(111) substrate, which was exposed to atomic hydrogen under UHV conditions to reduce the carbonyl (C = O) side groups. A subsequent annealing dehydrogenates these CH_2_ groups to CH radical sites as confirmed by AFM imaging. Combining tunneling spectroscopy and DFT + *U* calculations, we show by detecting a Kondo resonance in d*I*/d*V* spectra that single radical per monomer have a *S* = 1/2 spin state, which is accompanied by the introduction of localized low-energy states. Using DFT + *U* calculations, the influence of the number of radicals on the symmetry, magnetism, and electronic band structure of the KG is studied in details. We found that only the fully reduced KG structure restores the Dirac cones and Kagome flat bands and becomes metallic as they emerge near zero-energy, in contrast to its semiconducting counterpart. Although the experimental realization of the fully reduced KG structure still poses problems due to the limitations of the on-surface thermally activated dehydrogenation process, we now aim to utilize tip-induced dehydrogenation to complete the reaction and achieve the desired structure. Its synthesis in future experiments could open up opportunities to study correlated electronic phases in graphene such as magnetism or superconductivity.

Our results further demonstrate the potential of defect engineering to tune the electronic and magnetic properties of graphene materials for spintronics and quantum devices.

## Methods

### Sample Preparation

The Au(111) substrate purchased from Mateck GmbH was sputtered by Ar^+^ ions and annealed at 800 K to remove any surface contaminations. BRTANGO precursors were synthesized following the procedure described here.^33^ Molecules were sublimed from a Knudsen cell kept at 515 K in UHV. During sublimation, the sample was kept at about 470 K to promote the formation of well-extended Kagome graphene domains.

### Radical Formation Using Atomic Hydrogen

To reduce the carbonyl side groups, we used either a hydrogen cracker source from Focus GmbH or a home-built plasma source. Using the commercial cracking technique, atomic hydrogen are obtained by filling the chamber with a H_2_ gas to 2 × 10^–7^ mbar with a leak valve connected to the preparation chamber. A tungsten filament is heated to about 2770 K with an accelerating voltage of 1 keV until an emission power of 80 W was reached by the source as described here.^33^ The sample kept at room temperature was then placed in front of the source for a maximum of 4 min by opening its shutter. After exposure, the sample was immediately annealed to 470 K in UHV conditions to induce the radical’s formation. We repeated such preparation several times in order to increase the reaction yield since exposure times longer than 10 min led to the formation of small Au clusters on the surface, likely due to sputtering effects.

For the low-temperature plasma (LTP), we used a home-built source attached to the load lock chamber (Supporting Figure 4a). The sample hold at the center of the chamber on a wobble stick was grounded. The plasma was ignited at a hydrogen pressure of 1 × 10^–2^ mbars controlled by a pressure valve, which was then reduced to 2 × 10^–3^ mbars for the plasma treatment of the sample. LTP leads to a flux of ionized gas consisting of hot electrons, molecular and atomic ions, neutral species, and photons. The ion impact energy (about 20 eV) corresponds roughly to the difference between the plasma potential and the grounded sample. In order to avoid the creation of defects by the plasma (Supporting Figure 4), we exposed the backface of the sample using a typical plasma power of 20 W. This was followed by an annealing of the sample at 400 K in UHV to form radicals (Supporting Figure 5).

### STM Experiments

The STM experiments were conducted at a temperature of 4.8 K using an Omicron GmbH low-temperature STM/AFM system operated with Nanonis RC5 electronics. Differential conductance spectroscopy d*I*/d*V*(V) spectra were acquired with the lock-in amplifier technique by using a modulation of 610 Hz and a modulation amplitude of 10 meV. All voltages refer to the sample bias *V*_s_ with respect to the tip.

### AFM Experiments

AFM measurements were performed with commercially available tuning-fork sensors in the qPlus configuration^47^ equipped with a tungsten tip (*f*_0_ = 26 kHz, *Q* = 8000 to 25,000, nominal spring constant *k* = 1800 N m^–1^, oscillation amplitude A ≈ 50 pm). Constant-height AFM images were obtained using tips terminated with a single carbon monoxide (CO) in the noncontact mode (frequency-modulated AFM-FMAFM) at zero voltage.^21,22^ CO molecules were adsorbed on the sample maintained at low temperature below 20 K. Before its functionalization, the apex was sharpened by gentle indentations into the gold surface. A single CO molecule was carefully attached to the tip following the procedure of reference.^48^ Simulations of the AFM images based on the DFT coordinates were carried out using the probe-particle model.^42^ The Δ*f*(*V*) cross-section of 1 × 40 pixels^2^ was acquired with Au-coated metallic tips (tunneling set points: *I*_t_ = 1 pA, *V*_s_ = 200 mV, *Z*_offset_ = −50 pm).

### DFT Calculations

To investigate the electronic and magnetic properties of the 2D-KG with different number of defects, we employed the density functional theory (DFT) with the Hubbard correction (DFT + *U*) as implemented in the Quantum ESPRESSO package.^49,50^ This approach is necessary to account for the localized spin contributions and strong electron correlation effects within the 2D-KG lattice. We utilized the Perdew–Burke–Ernzerhof (PBE) functional^51^ in our DFT + *U* approach, which has been validated in our prior studies to yield accurate results for similar 2D systems.^8,52^ The Hubbard *U* parameter for the C 2p orbitals was derived using the linear response method,^53^ ensuring a consistent treatment of on-site electron–electron interactions. For geometry optimization of the TANGO 2D Kagome lattice, we employed an energy convergence threshold of 10^–6^ Ry and a force convergence criterion of 10^–4^ Ry/Å. A plane-wave cutoff energy of 50 Ry was used, and the Brillouin zone was sampled using a Monkhorst–Pack *k*-point grid of 6 × 6 × 1 for the unit cell optimization. For electronic structure and magnetic properties calculations, a denser *k*-point mesh of 12 × 12 × 1 was employed. A vacuum region of 20 Å was applied along the *z*-direction to avoid spurious interactions between periodic images, ensuring accurate representation of the 2D nature of the system. Spin-polarized calculations were performed to explore both antiferromagnetic (AF) and ferromagnetic (FM) coupling scenarios within the TANGO lattice, with spin alignments restricted along the *z*-axis for computational efficiency. The total magnetic moments and spin-density distribution were analyzed to explore the Jahn–Teller effects and magnetic couplings in the presence of multiple defects per unit cell (UC), as discussed in the main text. In addition to DFT + *U*, van der Waals (vdW) interactions were included using Grimme’s D2 method^54^ to capture weak interlayer interactions where applicable. Projector-augmented wave (PAW) pseudopotentials were used to describe the interaction between ions and valence electrons,^55,56^ ensuring accuracy in both the structural and electronic properties.

## Supporting Information Available

Self-assembly of BRTANGO molecules on Au(111) (Supporting Figure 1); force-voltage spectroscopy of the azatriangulene (Supporting Figure 2); high-resolution AFM images of reduced carbonyl groups (Supporting Figure 3); calibration of the plasma exposure (Supporting Figure 4); radical density versus plasma exposure time (Supporting Figure 5); spin-polarized DOS of a single radical (Supporting Figure 6); spin density maps of radical configurations (Supporting Figure 7); optimized structure in gas-phase of the unit well with six radicals (Supporting Figure 8); and nonspin-polarized band structure and PDOS of the fully reacted KG (Supporting Figure 9) (PDF)
