# Supplementary material for: On-Surface Synthesis and Characterization of Radical Spins in Kagome Graphene
Source: ACS Nano. 2025 Jan 10;19(4):4768–77. doi: 10.1021/acsnano.4c15519 (PMC11803911; doi:10.1021/acsnano.4c15519)
Supplement: Supplementary file 1 — nn4c15519_si_001.pdf [file nn4c15519_si_001.pdf]

# Supporting Information for : "On-Surface Synthesis and Characterization of Radical Spins in Kagome Graphene"


Rémy Pawlak,*,† Khalid N. Anindya,‡ Outhmane Chahib,† Jung-Ching Liu,† Paul Hiret,† Laurent Marot,† Vincent Luzet,¶ Frank Palmino,¶ Frédéric Chérioux,¶ Alain Rochefort,‡ and Ernst Meyer*,†

†*Department of Physics, University of Basel, Klingelbergstrasse 82, 4056 Basel, Switzerland*

‡*Engineering Physics Department, Polytechnique Montréal, Montréal (Québec), H3C 3A7, Canada*

¶*Université de Franche-Comté, FEMTO-ST, CNRS, F-25000 Besançon, France*

E-mail: remy.pawlak@unibas.ch; ernst.meyer@unibas.ch

# Self-Assembly of BRTANGO Molecules on Au(111)

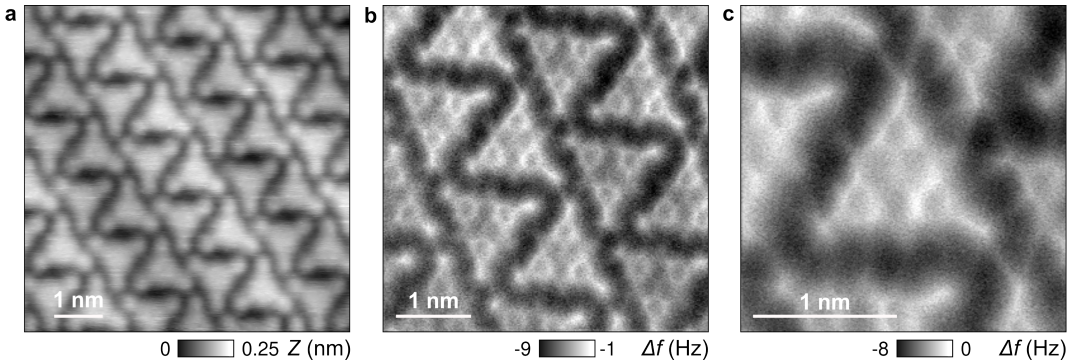


Supporting Figure 1: Self-assembly of BRTANGO molecules on Au(111). **a,** STM image of the BRTANGO self-assembly after deposition at room temperature, ($I_t$ = 1 pA, $V_s$ = 0.1 V). **b,** Corresponding AFM image with a CO-terminated tip showing the chemical structure of the BRTANGO assembly.

# Force-Voltage Spectroscopy of The Azatriangulene

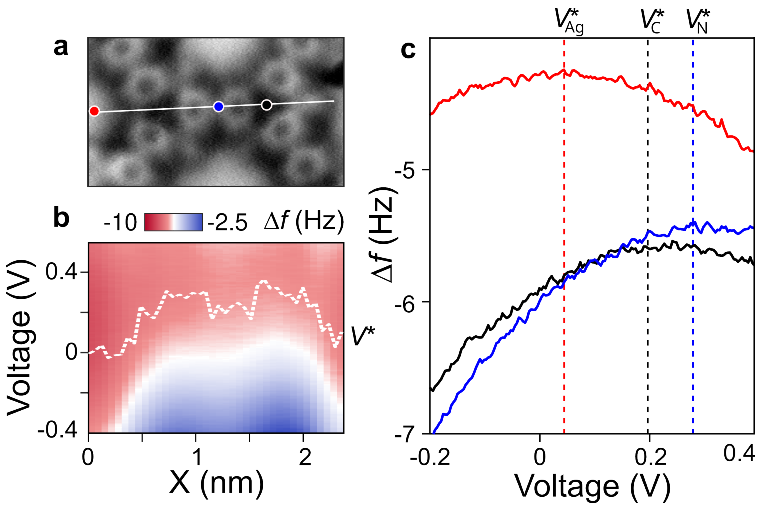


Supporting Figure 2: Force-voltage spectroscopy of the azatriangulene. **a**, AFM image of two azatriangulene monomers of the KG structure. **b**, $\Delta f(V, X)$ cross-section acquired along the white line of **e**. The black dashed line corresponds to the value of local contact potential difference ($V^*$). **c**, Representative $\Delta f(V)$ spectra acquired at the Au(111) (red), the azatriangulene (black) and a kagome segment (blue).

For the sake of completeness, we also investigate the charge distribution of the KG on Au(111) by force spectroscopy (Supporting Figure 2). By measuring the frequency shift $\Delta f$ as a function of the sample bias $V_s$ at a constant tip-sample separations $Z$ (see Experimental Section),[1–3] we probe the electrostatic force acting between tip and sample. As a result, $\Delta f(V)$ curves present a parabolic shape, in which the voltage corresponding to the top of the parabola (noted $V^*$) represents the local contact potential difference (LCPD) between tip and sample. Thus, the local variation of LCPD between site-dependent $\Delta f(V)$ curves allows us to estimate the charge distribution and local changes of work function down to the sub-molecular scale.[1–3] Supporting Figure S2b shows a $\Delta f(V)$ cross-section acquired along two azatriangulene monomers (white line in the AFM image of Supporting Figure 2a). Single $\Delta f(V)$ point-spectra acquired on top of Au(111) (red), at the central N atom (black) and between the monomers (blue) are plotted in Supporting Figure 2c, respectively. Dashed lines mark the LCPD value ($V^*$) for each position. The LCPD value systematically shifts towards more positive values ($\Delta V^* \approx 0.15$ and 0.23 V) for the KG structure as compared

to the pristine Au substrate, with a maximum value of 0.23 V for the central N atom of the azatriangulene. This indicates an accumulation of negative charges at the KG structure as compared to the Au substrate, which we attribute to a charge transfer from the substrate to the KG polymer due to its accepting character.

# High-Resolution AFM Images of Reduced Carbonyl Groups

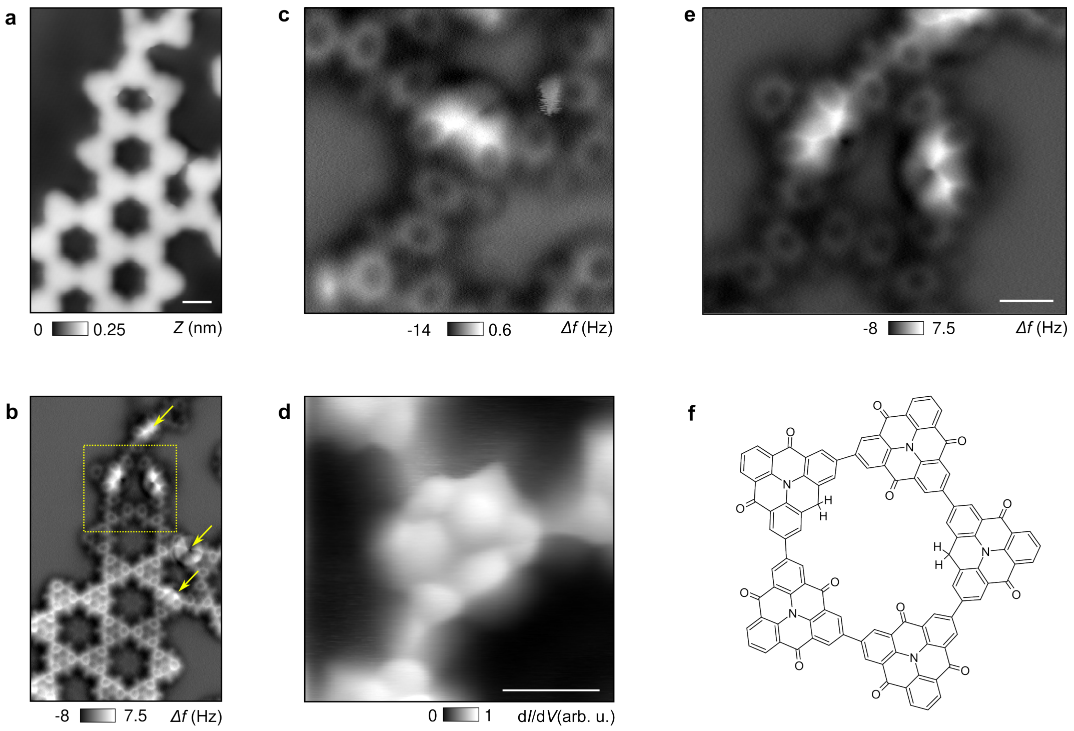


Supporting Figure 3: High-resolution imaging of reduced carbonyl groups. **a,** STM image of the kagome graphene exposed to atomic hydrogen, ($I_t$ = 1 pA, $V_s$ = 0.25 V). Scale bar is 1 nm. **b,** Corresponding AFM image with a CO-terminated tip showing the appearance of bright features at the side of few azatriangulene molecules (see yellow arrows). **c,** AFM image of a hydrogenated monomer and **d,** Corresponding BRSTM image. Scale bar is 0.5 nm. **e**, Close-up AFM image of partially hydrogenated monomers acquired in the yellow square of **c** and **f,** a tentative model of its chemical structure.

# Calibration of The Plasma Exposure

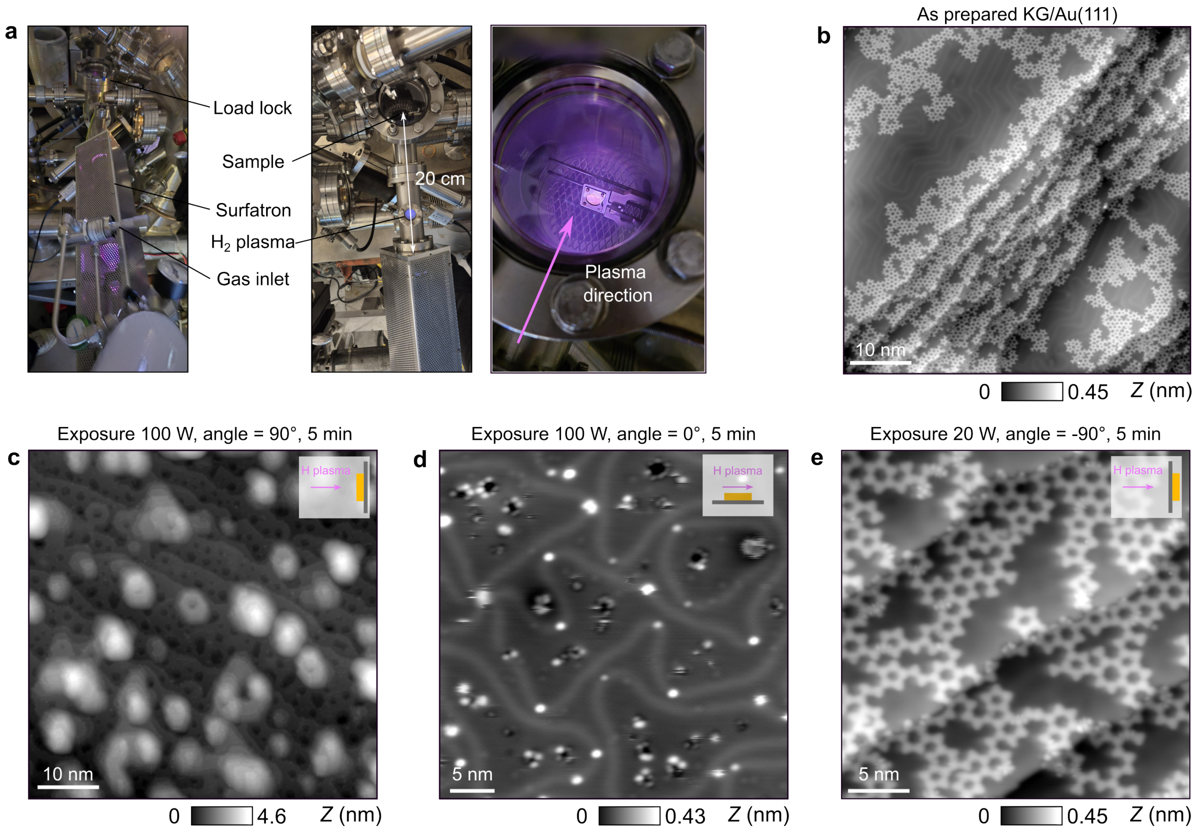


Supporting Figure 4: Effect of the hydrogen plasma on the KG/Au(111) sample. **a,** Picture of the plasma setup attached to the load lock chamber. **b,** Typical overview STM image showing the morphology of the KG/Au(111) before plasma treatment, ($I_t$ = 1 pA, $V_s$ = 0.1 V). **c,** STM image of the sample morphology after exposure for 5 minutes to the hydrogen plasma, ($I_t$ = 1 pA, $V_s$ = 0.1 V). Plasma was created 20 cm away from the sample in a Pyrex tube through a matching network by a 13.56 MHz radio frequency (RF) generator using a power (P) of 100W. The pressure during the discharge was $P$ $1{\times}10^{-2}$ mbar. This RF power was coupled to the tube by an outer electrode, the surfatron. The sample is facing the plasma flux (angle = 90°). **d,** STM image of the sample morphology after exposure for 5 minutes to the hydrogen plasma ($P$ = 100 W), ($I_t$ = 1 pA, $V_s$ = 0.1 V). The sample is in grazing incidence as compared to the plasma flux (angle = 0°). The plasma treatment etches the KG polymer. The gold surface does not show clusters but the herringbone reconstruction is slightly modified. **e,** STM image of the sample morphology after exposure for 5 minutes to the hydrogen plasma ($P$ = 20 W) and subsequent annealing of the substrate at 470 K. The sample is not directly exposed to the plasma direction (angle = -90°). Taking advantage of the atomic hydrogen produced in the plasma while avoiding the surface sputtering, this method leads to the reduction of KG carbonyl groups as described in the main manuscript.

Although numerous preparation procedures have been explored (in which we varied the

$H_2$ exposure time, the sample temperature and the number of cycles), we did not reach a reaction yield superior than 11-15 % using the hydrogen cracker.We noticed that longer exposure time to the hydrogen crackings source leads to the formation of Au clusters on the surface which we think to be due to sputtering by accelerated ions from the source.

To overcome this issue, we next employed a home-built plasma device as a source of atomic hydrogen (See Experimental Section). As shown in Supporting Figure 4a, the sample is hold at the center of the chamber on the wobble stick and grounded. The plasma is ignited at a pressure of $1\times10^{-2}$ mbar controlled by a pressure valve, which is then reduced to $2\times10^{-3}$ mbar for the plasma of the sample. The color of the plasma is then light purple. The distance between the plasma output and the sample is about 20 cm. In such hydrogen plasma, a small part contains atomic hydrogen while a large majority consists of ionized $H_2$ gas. Taking advantage of the atomic hydrogen produced in the plasma while avoiding ion sputtering (Supporting Figure 4), this method leads to the reduction of the KG carbonyl groups without any damages nor contamination of the surface (Supporting Figure 4e).

# Radical Density Versus Plasma Exposure Time

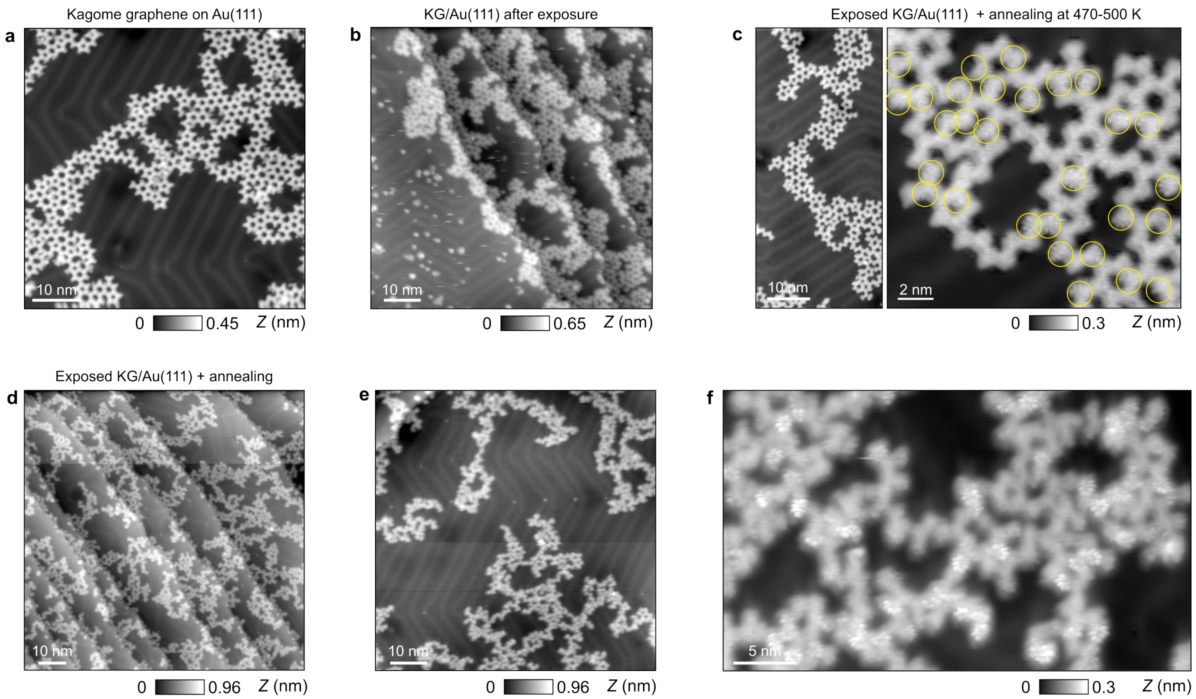


Supporting Figure 5: Radical density versus plasma exposure time. **a,** Typical overview STM image showing the morphology of the KG/Au(111) before plasma treatment. **b,** STM image of the KG polymer morphology after exposure for 5 minutes to the hydrogen plasma ($P$ = 20 W, angle = -90°). Aggregates adsorbed on terraces are contaminants obtained from the plasma exposure. **c,** STM images of the exposed KG/Au(111) shown in **b** after annealing the substrate to 470 K , ($I_t$ = 1 pA, $V_s$ = 10 mV). Contaminants are desorbed from the surface. The Au herringbone structure is intact demonstrating the non-invasive nature of the process. The right STM image shows the presence of radical sites in the KG polymer marked by yellow circles. **d-f,** Series of STM images of the KG/Au(111) exposed for 20 min in order to increase the reaction rate of the carbonyl reduction ($P$ = 20 W, angle = -90°), ($I_t$ = 1 pA, $V_s$ = 10 mV). The sample has been annealed to 470 K after exposure for all images. Whereas the number of radical sites has increased, the Kagome structure of the polymer is damaged by the plasma+annealing treatment.

Supporting Figure 5 shows the effect of exposure time on the KG reaction. We found that exposing the back-face of the Au surface to the plasma jet does not induce any damages of the surface. Just after exposure, few adsorbates are observed on Au terraces (Supporting Figure 4b) which we attribute to $H_2$ gas molecules. They are easily desorbed by annealing the surface to about 400 K for 10 minutes. As shown in Supporting Figure 5c, both the Au reconstruction and the KG lattice are intact after the plasma exposure and STM images show

the presence of radical sites (yellow circles). The number of reacted molecules (estimated from the STM image at low voltage) is about 40 %.

Note that we also increase the exposure to 20 minutes to reduce all carbonyl groups of the KG structure. As shown in the series of STM image (Supporting Figures 5d-f), all molecules are reacted as evidenced by a Kondo-like features in d$I$/d$V$ spectra but this reaction is always accompanied by the disruption of the Kagome lattice.

# Details of DFT calculations

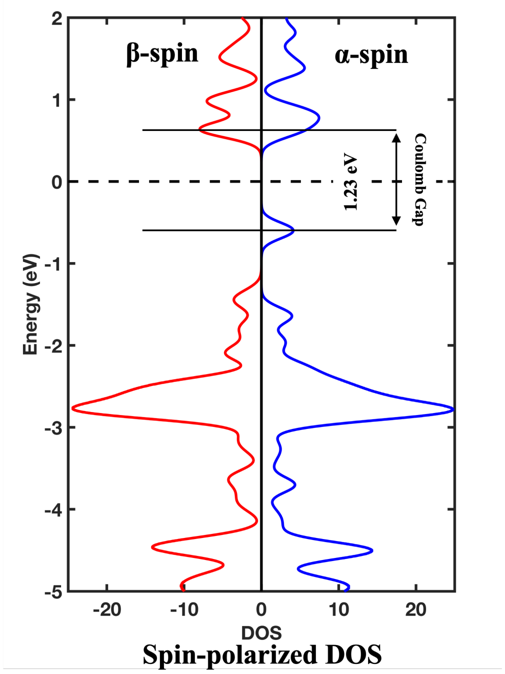


Supporting Figure 6: Spin-polarized DOS of a single radical. The spin-polarized DOS of one-radical per UC shows a distinct separation between the $\alpha$-spin and $\beta$-spin states, with a Coulomb gap of 1.23 eV. This gap reflects the alterations in the electronic structure caused by the unpaired electron present in the polymer.

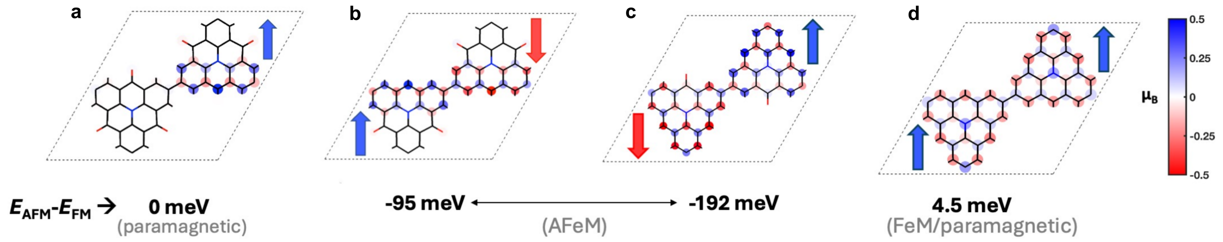


Supporting Figure 7: Spin density maps of radical configurations. **a-d**, Spin-density maps of the 2D-KG system with 1, 2, 4, and 6 radicals per unit cell, respectively. Energy differences ($E_{\mathrm{AF}} - E_{\mathrm{FM}}$) define the magnetic ground state stability. (**a**), The single-radical system is paramagnetic with 0 meV energy difference whereas (**b-c**), an antiferromagnetic (AF) coupling is observed in the two- and four-radical per UC systems with energy differences of -95 meV and -192 meV, respectively. (**d**), The fully reacted system shows ferromagnetic (FM) coupling with an energy difference of 4.5 meV. The Jahn–Teller distortion reduces the spin state to $S = 1/2$ in this configuration.

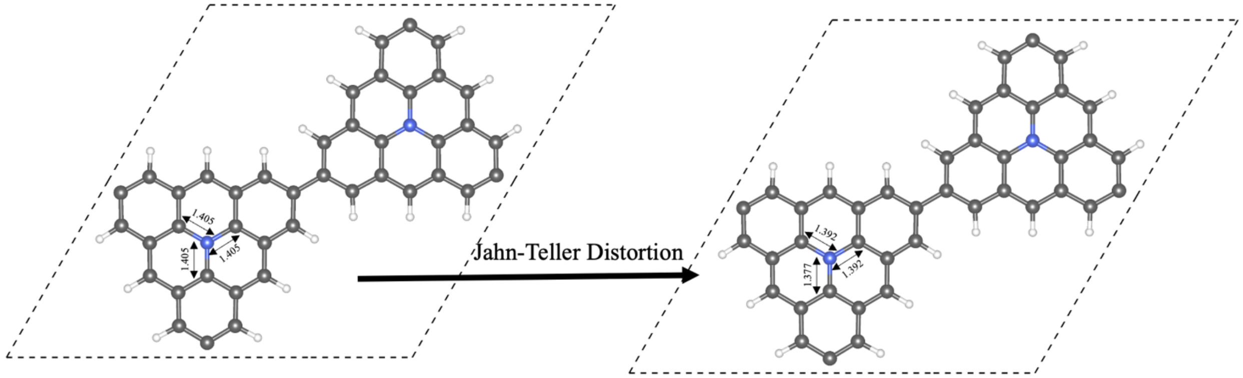


Supporting Figure 8: Optimized structure in gas-phase of the unit well with six radicals. A spin state of $S = 3/2$ exhibits a longer and equal bond length between the central N-C atoms of the azatriangulene backbone as compared to the structure optimized with spin state $S$ =1/2, which has shorter and non-equal bond lengths. Among these two resonant configurations, the Jahn-Teller distortion reduces the total energy of the $S = 1/2$ system, favoring it as compared to the $S = 3/2$ counterpart.

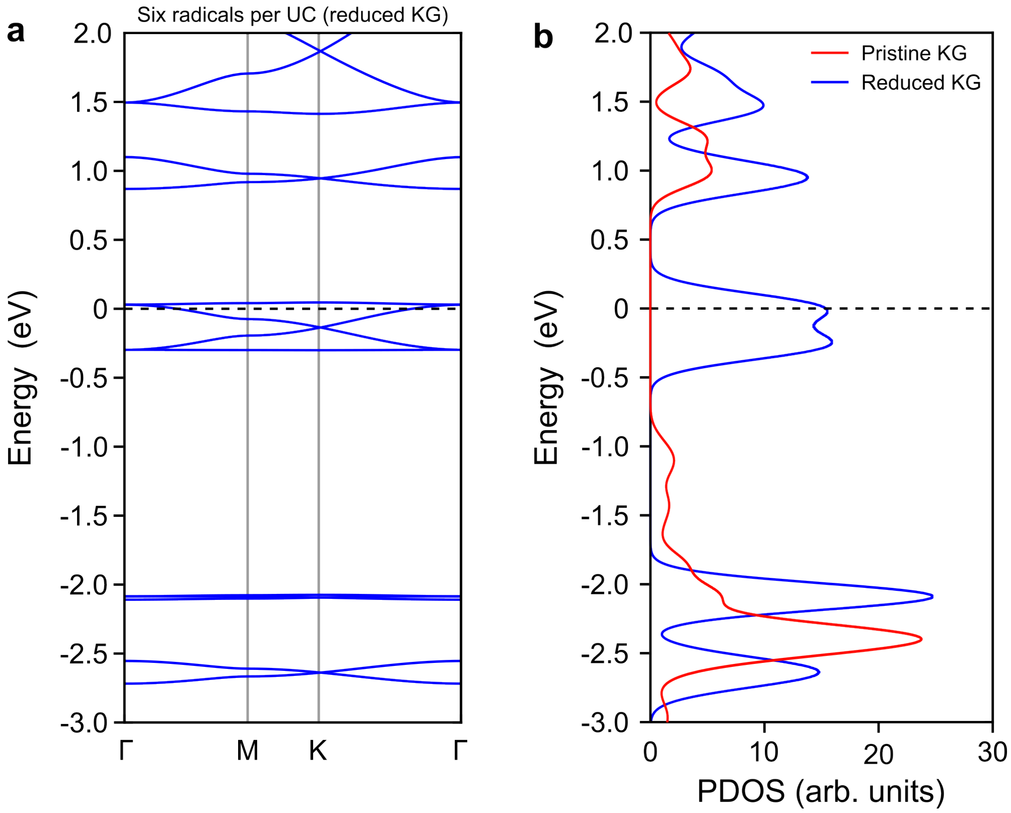


Supporting Figure 9: Non-spin polarized band structure and PDOS of the fully reacted KG. **a**, Calculated non-spin-polarized band structure for the freestanding the case of six radicals per UC (reduced KG). **b**, Non-spin-polarized density of states (DOS) calculated by DFT+$U$ of the six radicals per UC (blue) as compared to the pristine KG of Figure 2 (red). The near zero-mode states of the fully reacted KG is induced by the Dirac cone sandwiched by flat bands at $E_{\mathrm{F}}$ shown in **a**.

# References


(1) Mohn, F.; Gross, L.; Moll, N.; Meyer, G. Imaging the charge distribution within a single molecule. *Nat. Nanotechnol.* **2012**, *7*, 227–231.

(2) Meier, T.; Pawlak, R.; Kawai, S.; Geng, Y.; Liu, X.; Decurtins, S.; Hapala, P.; Baratoff, A.; Liu, S.-X.; Jelínek, P.; Meyer, E.; Glatzel, T. Donor–Acceptor Properties of a Single-Molecule Altered by On-Surface Complex Formation. *ACS Nano* **2017**, *11*, 8413–8420.

(3) Pawlak, R.; Sadeghi, A.; Jöhr, R.; Hinaut, A.; Meier, T.; Kawai, S.; Zajac, L.; Olszowski, P.; Godlewski, S.; Such, B.; Glatzel, T.; Goedecker, S.; Szymoński, M.; Meyer, E. Hydroxyl-Induced Partial Charge States of Single Porphyrins on Titania Rutile. *J. Phys. Chem. C* **2017**, *121*, 3607–3614.
